# Research Progress on Anti-Inflammatory Effects and Mechanisms of Alkaloids from Chinese Medical Herbs

**DOI:** 10.1155/2020/1303524

**Published:** 2020-03-18

**Authors:** Sicong Li, Xin Liu, Xiaoran Chen, Lei Bi

**Affiliations:** ^1^School of Pharmacy, Peking University Health Science Centre, Beijing, China; ^2^School of Traditional Chinese Medicine, Beijing University of Traditional Chinese Medicine, Beijing 100029, China

## Abstract

As the spectrum of diseases keeps changing and life pace keeps going faster, the probability and frequency of diseases caused by human inflammatory reactions also keep increasing. How to develop effective anti-inflammatory drugs has become the hotspot of researches. It has been found that alkaloids from Chinese medical herbs have anti-inflammatory, analgesic, antitumor, anticonvulsant, diuretic, and antiarrhythmic effects, among which the anti-inflammatory effect is very prominent and commonly used in the treatment of rheumatoid arthritis, ankylosing spondylitis, and other rheumatic immune diseases, but its mechanism of action has not been well explained. Based on this, this paper will classify alkaloids according to structural types and review the plant sources, applicable diseases, and anti-inflammatory mechanisms of 16 kinds of alkaloids commonly used in clinical treatment, such as berberine, tetrandrine, and stephanine, with the aim of providing a reference for drug researches and clinical applications.

## 1. Introduction

Inflammation has been considered as the main risk factor of rheumatic immune diseases. As the incidence rate of this disease keeps increasing in recent years, how to effectively resist inflammation has attracted widespread attentions. The application of anti-inflammatory Chinese medical herbs not only enjoys a long history, a wide range of sources, and rich varieties, but also has complex and diverse pharmacological effects. Therefore, it has become a research hotspot to search for anti-inflammatory active ingredients in Chinese medical herbs, among which alkaloids are of great representativeness. Alkaloids are nitrogen-containing organic compounds with an alkali like properties. They are widely distributed in *Tripterygium wilfordii* and *Begonia kunmingshanensis* of Celastraceae; *Sinomenium acutum*, *Stephania tetrandra* S. Moore, and *Menispermum dauricum* DC. of Menispermaceae [1]; *Coptis chinensis* Franch., *Aconitum carmichaelii*, *Aconitum kusnezoffii* Reichb., *Aconitum carmichaelii* Debx., and *Clematis chinensis* Osbeck of Ranunculaceae; *Gentiana macrophylla* Pall. and *Gentiana scabra Bunge*. of Gentianaceae, and so on [2]. Some alkaloids have strong anti-inflammatory activities and play important roles in the treatment of rheumatoid arthritis, Behcet's disease, ankylosing spondylitis, myasthenia gravis, systemic lupus erythematosus, dermatomyositis, and other rheumatic immune diseases [3]. The following is a comprehensive review of researches on alkaloids from Chinese medical herbs. By taking chemical structure as the classification standard, anti-inflammatory effects and possible mechanisms of various alkaloids are introduced in detail, which are expected to lay the foundation for follow-up researches and developments of anti-inflammatory drugs.

## 2. Isoquinoline Alkaloids

Isoquinoline alkaloids are widely distributed in 27 sections of plants, such as Menispermaceae, Berberidaceae, Papaveraceae, and Ranunculaceae [4]. Isoquinoline alkaloids, taking isoquinoline or tetrahydroisoquinoline as the basic parent nucleus, can be divided into 20 categories, including simple isoquinoline, benzylisoquinoline, phenethyl isoquinoline, naphthyl isoquinoline, aporphine, morphine, phenanthridine, and pyrrolidine. Isoquinoline alkaloids have anti-inflammatory, analgesic, spasmolysis, antibacterial, and relieving asthma effects [5]. Among them, berberine, tetrandrine, dauricine, sinomenine, lycorine, and stephanine have good anti-inflammatory effects.

### 2.1. Berberine

Berberine, a kind of benzylisoquinoline alkaloid extracted from the rhizome of *Coptis chinensis* Franch., the bark of *Phellodendron chinensis* Schneid., and *Daemonorops margaritae* (Hance) Becc. [6], has therapeutic effects on rheumatoid arthritis, delayed type hypersensitivity, ulcerative colitis, autoimmune tubulointerstitial nephritis, and other rheumatic autoimmune diseases [7]. 8 mg/kg of berberine subcutaneously injected to rats can inhibit rats' paw swelling induced by carrageenan and mice's auricle swelling induced by xylene. Intraperitoneal injection of berberine (50 mg/kg) can inhibit the increased skin capillary permeability caused by histamine [8].

Intraperitoneal injection of 30 mg/kg of berberine for 8 days can inhibit the increased prostaglandin E2 (PGE2) in paw swelling mice induced by formalin [9]. The mechanism is that berberine can inhibit PGE2 synthesis by reducing the concentration of cyclooxygenase-2 (COX-2). Oral administration of berberine (1.5 mg/ml) can inhibit the delayed hypersensitivity induced by dinitrofluorobenzene in mice [10], by means of inhibiting the production and secretion of interferon-*γ*, interleukin-1 (IL-1), tumor necrosis factor (TNF-*α*), interleukin-2 (IL-2), interleukin-8 (IL-8), interleukin-6 (IL-6), and monocyte chemotactic protein-1 (MCP-1) [11]. Berberine has a therapeutic effect on autoimmune nephritis of rats induced by alloantigens, such as primary anti-glomerular basement membrane and Heymann nephritis. It can inhibit the excretion of urinary protein and reduce serum creatinine content and pathological changes of glomerulus [12].

In terms of diseases in the digestive system, berberine can protect the gastric mucosa from chemical factors. Berberine can inhibit the activation of nuclear factor kappa-B (NF-*κ*B) and the transcription of inflammatory cytokines in ulcerative colitis mice induced by dextran sodium sulfate [13]. Berberine can inhibit not only the adhesion between endothelial cells and polymorphonuclear leukocytes activated by IL-1 and tumor necrosis factor-*α* (TNF-*α*) but also the adhesion of polymorphonuclear leukocytes and vascular endothelial cells activated by TNF-*α*, so as to reduce the infiltration of inflammatory cells and lesions of colonic mucosa [14].

### 2.2. Tetrandrine

Tetrandrine is a kind of bisbenzylisoquinoline alkaloid extracted from the root of *Stephania tetrandra* S. Moore. and *Stephania japonica* (Thumb.) Miers [15]. It has therapeutic effects on gout, osteoarthritis, and systemic lupus erythematosus. Tetrandrine can significantly inhibit rats' joint swelling caused by carrageenan or formaldehyde and increased vascular permeability in rats induced by histamine [16]. Intraperitoneal injection of 20 mg/kg of tetrandrine can significantly reduce the amount of hydrothorax, protein exudation, and leukocyte migration in rats with pleurisy caused by carrageenan [17]. The effect of inhibiting leukocyte migration is stronger than the effect of inhibiting the exudation of hydrothorax and proteins. Intraperitoneal injection of 20 mg/kg of tetrandrine also significantly reduces the activity of neutrophils (Neu-PLA2) and the acellular component (ACC-PLA2) [18]. Tetrandrine in the treatment of experimental auricular burns plays a direct excitatory adrenal function. Tetrandrine not only inhibits cyclooxygenase (COX)-2 and nitric oxide synthase (iNOS) by interfering with NF-*κ*B/COX-2 signaling pathway, but also inhibits histamine, platelet-derived growth factor (PDGF), IL-1*β*, and TNF-*α* in inflammatory reactions and promotes the expression of the anti-inflammatory cytokine IL-10 [19].

Intragastric administration of 6.25 mg/kg of tetrandrine can reduce the concentration of nitric oxide in serum and pancreatic tissue of rats with acute hemorrhagic necrotizing pancreatitis, reduce the activity of phospholipase A2, and inhibit the activation of NF-*κ*B, thus inhibiting the production of IL-6, IL-8, and TNF-*α* inflammatory factors, as well as reducing the inflammatory response of pancreas [20].

In the inflammatory model of RAW264.7 cells induced by lipopolysaccharide (LPS), tetrandrine can not only inhibit the phosphorylation of NF-*κ*B inhibitor *α*, but also reduce the production of nitric oxide (NO) and prostaglandin E2 (PGE2), and the expression of matrix metalloproteinase-3 (MMP-3) and tissue metalloproteinase inhibitor-1 (TIMP-1) [21].

### 2.3. Dauricine

Dauricine, a kind of benzylisoquinoline alkaloid extracted from the rhizome and rattan of *Menispermum dauricum DC* [22], has therapeutic effects on rheumatoid arthritis, ischemic stroke, coronary atherosclerotic heart disease, and ventricular premature beat.

Dauricine can significantly improve neurological deficit symptoms and reduce the apoptosis rate of neurons in ischemic brain tissue [23]. Moreover, it can inhibit the expression of nuclear factor КB, intercellular adhesion molecule-1, and cyclooxygenase-2. Intraperitoneal injection of 40 mg/kg of dauricine can effectively reduce liver injury in mice induced by CCl_4_. Its mechanism is related to inhibiting the expression of TGF *β*1 and TNF-*α* [24].

In vitro studies, it is identified that pretreatment of dauricine can dose-dependently inhibit not only the contents of NO, IL-1*β*, IL-6, TNF-*α*, and intercellular adhesion molecule-1 (ICAM-1), but also the activity of inducible nitric oxide synthase (iNOS), myeloperoxidase (MPO), and cyclooxygenase-2 (COX-2) in LPS-stimulated macrophages [25].

Intraperitoneal injection of 50 mg/kg of dauricine, with pharmacological actions similar to hydrocortisone, can inhibit the increased capillary permeability caused by histamine and acetic acid, inflammatory edema caused by xylene in mice, and edema of hind paws caused by carrageenan in rats [26]. Dauricine has the same anti-inflammatory effect on adrenalectomized rats. The anti-inflammatory effect of dauricine at the same dosage in normal rats is stronger than that in adrenalectomized rats. Dauricine can also reduce the content of ascorbic acid in adrenal glands. It can be concluded that dauricine also has an indirect anti-inflammatory effect by stimulating the pituitary adrenal cortex system [27].

### 2.4. Sinomenine

Sinomenine, a kind of benzyl alkaloid, is extracted from the stem and root of *Sinomenium acutum* (Thunb.) Rehd. et Wils. [28]. It can be used in the treatment of rheumatoid arthritis, chronic nephritis, ankylosing spondylitis, myocardial ischemia, ventricular premature beats, and other rapid arrhythmias. Sinomenine can inhibit the foot swelling caused by egg white, formaldehyde, or carrageenan [29].

Oral administration of 30 mg/kg of sinomenine can not only inhibit the activity of iNOS and COX-2 in rats but also inhibit the synthesis and release of PGE2 and leukotriene in inflammatory areas. Sinomenine can alleviate LPS-induced lung inflammation by inhibiting the expression of nitric oxide (NO), myeloperoxidase (MPO), TNF-*α*, and IL-6 [30].

Sinomenine achieves the anti-inflammatory effect by inhibiting NF-*κ*B and JNK signaling pathways. The mechanism is that it inhibits the expression of TNF-*α* mRNA and IL-1*β* mRNA in synovial cells by inhibiting NF-*κ*B activity [31]. Sinomenine can not only inhibit the synthesis of prostaglandin F2a (PGF2a) and leukotriene in macrophages, but also reduce the synthesis of nitric oxide (NO) in cells [32]. Sinomenine can prevent the aggregation of neutrophils caused by complement activation, so as to improve rat antigenic arthritis caused by calf serum albumin. It is found that sinomenine can inhibit the proliferation of lymphocytes induced by mitosis and mixed lymphocyte culture. At the same time, the concentration of IL-1, IL-6, TNF, and other inflammatory cytokines in the supernatant of cultured cells decreases [33]. Moreover, sinomenine has the ability to inhibit inflammation in LPS-stimulated macrophages by regulating CD14/TLR4, JAK2/STAT3 pathway, and calcium signal through alpha 7nAChR [34].

### 2.5. Lycorine

Lycorine, a kind of pyrrolidine alkaloid extracted from the bulb of *Lycoris radiata* (L′ Her.) Herb., has a therapeutic effect on osteoarthritis, deformable osteitis, gout, ankylosing spondylitis, and other rheumatic autoimmune diseases. Intravenous injection of 3 mg/kg of lycorine has a therapeutic effect on formalin arthritis in rabbits and protein arthritis in rats [35]. In the endotoxic shock model mice, 80% of the mice in normal saline group die of endotoxic shock within 40 hours after injecting LPS. After intravenous injection of 40 mg/kg of lycorine, the survival rate of mice gets to 60%, rather than 20% in the normal saline control group [36].

Tolerance ability of inflammatory factors in mice gets improved after injecting lycorine. It is found that lycorine (5 *μ*m) can inhibit activities of iNOS and cyclooxygenase-2 induced by LPS. The inhibitory effect is stronger than that of dexamethasone. In addition, lycorine can also inhibit the production of PGE2 and IL-6 in macrophages to play an antipyretic and analgesic role [37].

A STAT signaling pathway is one of the common pathways of physiological and pathological reactions in the human body. JAK-STAT  pathway gets involved in the signal transduction and regulation of many important inflammatory/anti-inflammatory cytokines [38]. STATs are many cytoplasmic proteins that bind to the DNA of the target gene regulatory region. They are conjugated to tyrosine phosphorylation signals to complete transcriptional regulation. It is found that lycorine (5 *μ*M) inhibited the activation of STAT1 and STAT3 stimulated by LPS [39].

### 2.6. Stephanine

Stephanine, a kind of simple isoquinoline alkaloid, is extracted from the root and tuber of *Menispermum japonicum* Thunb. [40]. It has a therapeutic effect on rheumatoid arthritis, osteoarthritis, and gout. Stephanine has an obvious inhibitory effect on xylene induced inflammatory edema, toe swelling induced by egg white, and adjuvant arthritis. Intraperitoneal injection of 10 mg/kg of stephanine can reduce myeloperoxidase (MPO) activity and contents of TNF-*α*, IL-1*β*, and IL-6 in the mammary gland tissue of mastitis rats induced by LPS [41]. Stephanine at this dosage can not only inhibit p65 phosphorylation and I*κ*B degradation, but also improve the pathological changes of breast tissue. Intraperitoneal injection of 20 mg/kg of stephanine can reduce the expression of NF-*κ*B, phospho-p38 MAPK, phospho-JNK, NLRP3, and IL-1*β* in the brain tissue of mice with focal cerebral ischemia, so as to play an anti-inflammatory role [42]. Intragastric administration of 15 g/kg of stephanine can not only reduce the content of ICAM-1, IL-1*β*, and IL-18 but also increase the content of IL-12 in peripheral blood and nasal lavage fluid [43].

## 3. Piperidine Alkaloids

Piperidine alkaloids are derived from lysine, including piperidine, indolicidin, and quinolizidine. These alkaloids can be found in *Sophora flavescens* Ait., *Diphasiastrum veitchii.*, *Piper nigrum* L., *Lobelia chinensis* Lour., etc. [44]. Piperidine alkaloids have anti-inflammatory, anticonvulsant, anticancer, and antiarrhythmic effects. Among them, aloperine, sophoridine, and matrine have good anti-inflammatory effects [1].

### 3.1. Aloperine

Aloperine, extracted from *Sophora alopecuroides*, has therapeutic effects on myocardial ischemia-reperfusion injury, acute renal injury, asthma, ventricular hypertrophy, pulmonary hypertension, and other cardiovascular diseases [45]. The concentration of IL-6, TNF-*α*, and IL-1*β* in H9c2 cardiomyocytes decreases significantly after intragastric administration of 50 mg/L of aloperine for 48 hours, which suggests that aloperine can alleviate myocardial ischemia-reperfusion injury by inhibiting inflammatory response [46]. Intragastric administration of 40 mg/kg of aloperine for 14 days can reduce the levels of IL-6, IL-1*β*, TNF-*α*, NO, MCP-1, ADMA, ICAM-1, and VCAM-1, thus inhibiting the myocardial fibrosis and ventricular hypertrophy caused by isoproterenol [47].

IL-6 and TGF-*β*1 play an important role in the development of pulmonary hypertension. Pulmonary hypertension is often accompanied by a large number of inflammatory cells infiltration into pulmonary vascular lesions. The expression levels of NF-*κ*B, TNF-*α*, and IL-1*β* protein significantly reduce after intragastric administration of 100 mg/kg of aloperine [48]. The result of flow cytometry shows that 0.5 mM of aloperine can reduce the expression levels of NF-*κ*, Bp65, p-IKK*α*, p-I*κ*B*α*, TNF-*α*, and CyclinE1 [49]. After the application of aloperine, the injury of pulmonary vascular endothelial cells gets significantly improved, the shape of the nucleus of endothelial cells becomes basically normal, and the expansion of the Golgi body and endoplasmic reticulum gets improved. It suggests that rotenone can improve pulmonary hypertension induced by monocrotaline through the NF-*κ*B p65 signaling pathway [50].

In terms of respiratory diseases, intragastric administration of 40 mg/kg of aloperine can improve lung function, alleviate asthma symptoms, and inhibit inflammatory responses in asthmatic mice. The mechanism is related to the regulation of the NF-*κ*B inflammatory signal pathway and the reduction of TNF-*α* and IL-1*β* levels [51].

### 3.2. Sophoridine

Sophoridine, extracted from the roots of *Sophora flavescens*, has therapeutic effects on rheumatoid arthritis and ankylosing spondylitis, as well as organ damage caused by endotoxin and exogenous toxicant [52]. Intragastric administration of 40 mg/kg of sophoridine can reduce the increased permeability of capillaries and inhibit the auricle swelling induced by xylene [53]. Intraperitoneal injection of 5 mg/kg of sophoridine for 3 days in advance can prevent the lung and kidney injury caused by endotoxin and reduce the congestion, edema, and blood seepage into lung tissue as well as edema and inflammatory cell infiltration in renal tubular epithelial cells [54]. Its anti-inflammatory mechanism is to downregulate the expression of lipopolysaccharide recognition receptor, lipopolysaccharide-binding protein, CD14, and TLR4, as well as the transcription of nuclear factor c-Jun and c-fos genes. Sophoridine inhibits not only the phosphorylation of p38 mitogen-activated protein kinase (p38 MAPK) but also the expression and release of IL-6, TNF-*α*, and NO [55]. Continuously intraperitoneal injection of 125 mg/kg of sophoridine for 8 weeks significantly inhibits acute liver injury in mice induced by carbon tetrachloride [56]. The damage of liver tissue in mice gets alleviated, which appears as the orderly arrangement of hepatocytes, the reduction of focal necrosis, cell balloon degeneration, steatosis, and interstitial inflammation. The protective mechanism of sophoridine is related to its inhibition of cyclooxygenase-2 activity and the reduction of TNF-*α* and other inflammatory cytokines [57].

### 3.3. Matrine

Matrine, extracted from the root of *Sophora flavescens*, has therapeutic effects on rheumatoid arthritis, psoriasis, ulcerative colitis, ankylosing spondylitis, asthma, acute lung injury, acute respiratory distress syndrome, and cerebral infarction [58].

Hypodermic injection of 25 mg/kg of marine can inhibit the swelling of auricle caused by croton oil, the acute inflammatory swelling of the toes caused by carrageenan, or egg white [59]. Moreover, it can inhibit the increased capillary permeability in the abdominal cavity caused by acetic acid in both normal mice and adrenalectomized mice.

In terms of respiratory diseases, 3 hours after endotoxin injection, intraperitoneal injection of marine (10 mg/kg) can resist the pathological changes of lung tissue in mice caused by lipopolysaccharide and reduce pulmonary edema, pulmonary vascular leakage, and rise in temperature [60]. Moreover, it can not only inhibit the activity of myeloperoxidase (MPO) and malondialdehyde (MDA) but also reduce the content of TNF-*α*, IL-1*β*, and IL-6. Matrine can inhibit airway inflammation by inhibiting the signal transduction of NF-*κ*B in airway epithelial cells of asthmatic mice, reducing the expression of SOCS3 as well as the production of reactive oxygen species (ROS) and inflammatory factors in alveolar macrophages [61].

Matrine also has a protective effect on lung injury caused by ulcerative colitis. The mechanism is to enhance the anti-inflammatory and scavenging oxygen free radical ability of lung tissue and upregulate the repair factors in lung and gut tissue by increasing the expression of ZO-1 and Occludin, which are closely related to the mucosa of lung tissue. Matrine has a strong inhibitory effect on TNF-*α* and can effectively reduce the secretion of IL-1*α* and IL-8 [62].

In terms of nervous system diseases, injecting matrine (30mg/kg) into the jugular vein can inhibit the activity of 12/15-lipoxygenase and the expression of NF-*κ*B, TLR4, and TLR2 in ischemic brain tissue [63]. Moreover, it can reduce not only the nerve function defect of right middle cerebral artery occlusion model rats but also the volume of local cerebral infarction. The results above suggest that matrine has an obvious neuroprotective effect on ischemic brain tissues.

In vitro, matrine at the dosage of 50 *μ*mol/L can significantly inhibit the expression of IL-6, IL-8, TNF-*α*, NF-*κ*B, and intracellular adhesion molecule 1 in colon epithelial cells induced by lipopolysaccharide. Matrine at the dosage of 100 *μ*mol/L can significantly resist the inflammatory damage of colon epithelial cells induced by lipopolysaccharide [64].

## 4. Terpene Alkaloids

Terpene alkaloids come from mevalonic acid. They can be classified into four categories, which include monoterpenes, sesquiterpenes, diterpenes, and triterpenes. Terpene alkaloids have antihypertensive, analgesic, anti-inflammatory, antipyretic, and sedative effects. Among them, gentianine, aconitine, and bulleyaconitine A have good anti-inflammatory effects [65].

### 4.1. Gentianine

Gentianine, a kind of monoterpene alkaloid, is extracted from the root of *Gentiana scabra* Bunge., the whole herb of *Gentiana algida* Pall., and the seeds from *Trigonella foenum-graecum*. Intraperitoneal injection of 90 mg/kg of gentianine for 10 days can reduce not only the increase of capillary permeability caused by egg white but also the formaldehyde-induced foot swelling in rats, accelerating the swelling to subside as well [66]. The effect is equivalent to 200 mg/kg of sodium salicylate but does not lead to gastrointestinal bleeding [67]. The content of vitamin C in adrenal gland of rats significantly decreases after injecting gentianine. But this effect disappears after the removal of the pituitary gland. Therefore, it can be concluded that the anti-inflammatory effect of gentianine is related to hypophyseal-adrenocortical functions.

Gentianine inhibits IL-1*β*-induced inflammatory responses in rats' articular chondrocytes by inhibiting the activity of P38, extracellular regulated protein kinases (ERK), and c-Jun N-terminal kinase (JNK). In addition, gentianine can also inhibit the release of matrix metalloproteinases (MMPs) induced by IL-1*β* and promote the expression of type II collagen [68]. Oral administration of 50 mg/kg of gentianine for 12 weeks can reduce body weight and visceral fat mass in mice, which was associated with reduced levels of inflammatory cytokines (NF-*κ*b1, TNF-*α*, and IL-6). Oral administration of gentianine at the dosage of 60 mg/kg for 7 days has a therapeutic effect on contact dermatitis induced by 1-fluoro-2,4-dinitrofluorobenzene (DNFB) [69]. Gentianine can inhibit epidermal hyperplasia and immune cell infiltration, as well as reducing the production of TNF-*α*, IFN-*α*, IL-6, and MCP-1 in inflammatory tissues.

### 4.2. Aconitine

Aconitine, a kind of diterpene alkaloids extracted from the root tuber of *Aconitum carmichaeli* Debx., has a therapeutic effect on rheumatoid arthritis, ankylosing spondylitis, and other autoimmune diseases [70]. Oral administration, subcutaneous injection, or intramuscular injection of aconitine can significantly reduce the content of ascorbic acid in the adrenal glands of rats. This effect cannot be blocked by pentobarbital and chlorpromazine. Oral administration of 0.1 mg/kg of aconitine has inhibitory effects on the edema of toes in rats caused by carrageenan, ear swelling in mice caused by croton oil, and joint swelling in rats caused by formaldehyde [71]. The effect of aconitine exerted on the symptoms above is stronger than indometacin. Intragastric administration of 60 mg/L of aconitine can improve the acute lung injury in rats caused by lipopolysaccharide, by means of inhibiting the activation of NF-*κ*B, reducing the contents of inflammation transcription factor NF-*κ*B and inflammatory mediators TNF-*α*, IL-6, and IL-1*β*. Moreover, no evidence has been founded to support aconitine's toxicity to the cells of the lung tissue [72].

### 4.3. Bulleyaconitine A

Bulleyaconitine A, a kind of aconitine diterpenoid alkaloids, is extracted from the root tuber of *Aconitum kusnezoffii* Rchb. [73]. Nowadays, bulleyaconitine A has been widely used in the clinical treatment of rheumatoid arthritis, ankylosing spondylitis, knee osteoarthritis, and gout. Bulleyaconitine A has an obvious anti-inflammatory effect by inhibiting the phagocytic function of macrophages as well as the release of nitric oxide, INF-*γ*, and PGE2 [74]. Intraperitoneal injection of 0.48 mg/kg for 7 consecutive days can effectively reduce the content of IL-4, IL-10, IL-6, TNF-*α*, and MCP-1 produced by LPS-induced RAW264.7 cells [75]. Intraperitoneal injection of 0.48 mg/kg can not only decrease the expression of NF-*κ*B1 and PKC-*δ* in the NF-*κ*B signaling pathway in the lung tissues of asthmatic mice, but also reduce the contents of IL-4, IL-10, TNF-*α*, and MCP-1 in bronchoalveolar lavage fluid [76].

## 5. Purine Alkaloids

Purine alkaloids have the same purine ring structure and can be transformed into each other under specific conditions, mainly including caffeine, theophylline, theobromine, xanthine, and hypoxanthine. Among them, theophylline has a good anti-inflammatory effect and has been widely used in the clinical treatment of Chronic Obstructive Pulmonary Disease (COPD) and other pulmonary diseases.

### 5.1. Theophylline

Theophylline, extracted from tea and cocoa beans, has the functions of anti-inflammatory, bronchiectasis, immunity regulation, and improving the contractility of septal muscle. It has therapeutic effects on bronchial asthma, emphysema, and heart failure [77]. Theophylline exerts anti-inflammatory effects by inhibiting the release of phosphodiesterase and neuropeptides. At appropriate levels, theophylline strongly inhibits antigen-activated late-phase reactions, which are associated with the inhibition of the activation of neutrophils and the release of inflammatory mediators induced by phosphodiesterase. Theophylline has direct dilating effects on the smooth muscle of the respiratory tract [78]. Theophylline also inhibits adenosine receptor, increases the release of interleukin-10, and inhibits the transcriptional expression of NF-*κ*B and the activation of inflammatory cells and T cells from peripheral blood to airway mucosa metastasis, thus playing an anti-inflammatory and immune regulatory role. Oral administration of 0.1 g of theophylline twice a day can reduce the contents of IL-4, IL-5, IL-6, IL-8, IL-17, TNF-*α*, and C-reactive protein (CRP) in COPD patients. Moreover, theophylline of this dosage can increase the activity and expression of histone deacetylase-2 in the blood of COPD patients and improve the sensitivity of the human body to glucocorticoids [79].

Moreover, a low dosage of theophylline can inhibit the abnormal expression of ET-1 induced by inflammatory cytokines in smooth muscle, which is one of its mechanisms of reducing the hyperresponsiveness of asthmatic airway.

## 6. Organic Amine Alkaloids

Organic amine alkaloid is a kind of important alkaloid. Nitrogen atoms in this alkaloid are not combined in a ring structure. Colchicine, betaine, ephedrine, demecolcine, and leonurine belong to this kind of alkaloids.

### 6.1. Colchicine

Colchicine, extracted from the seeds and bulbs of *Colchicum autumnale* L., has been now widely used in the treatment of gout and the first choice for acute gout attacks. The 2016 European rheumatic union guidelines for the treatment of gout [80] suggests that colchicine is almost equivalent to nonsteroidal anti-inflammatory drugs and glucocorticoids and the guidelines advocates to start the treatment of gout with low doses of colchicine.

Monosodium urate crystal deposition is the central link in the pathogenesis of gouty arthritis. When neutrophils phagocytize monosodium urate crystals, lysosomes can be induced to release a chemokine derived from glycopeptide crystals, which can significantly amplify the recruitment of neutrophils. Colchicine, by means of inhibiting the aggregation of neutrophils at the joints, weakens neutrophils' phagocytosis towards monosodium urate crystals and reduces the inflammatory response caused by partial neutrophils [81]. Moreover, colchicine can change the expression of L-selectin in neutrophils and the distribution of E-selectin in endothelial cells, so as to inhibit the release of leukotriene B4, as well as the adhesion, exudation, and recruitment of neutrophils [82]. Colchicine can inhibit not only the chemotaxis, adhesion, and superoxidation of neutrophils but also the process and release of NOD-like receptor heat protein domain-related protein inflammasomes and interleukin-1*β*.

In terms of heart disease, oral administration of 1.5 mg of colchicine for 5 days can reduce hypersensitive C-reactive protein (hs-CRP), IL-1, IL-6, IL-8, and Neutrophilic Alkaline Phosphatase 3 (NALP3) levels in patients' serum with acute myocardial infarction [83]. Moreover, it can not only improve the expression of IL-1, apoptosis-related microprotein ASC, cysteine, proteinase-caspase-1, and NLRP3 mRNA in peripheral blood but also reduce the myocardial damage mediated by the inflammatory response. Colchicine can prevent the recurrence of atrial fibrillation after radiofrequency ablation, which is closely related to the reduction of the contents of interleukin-6 and CRP. Vascular atherosclerotic disease is a chronic immune inflammatory disease and smoking is an independent risk factor for atherosclerosis disease. After the oral administration of colchicine (0.1 mg/(kg·d)) for eight weeks, the contents of intercellular adhesion molecule-1 (ICAM-1), vascular adhesion molecules-1 (VCAM-1), monocyte chemoattractant protein-1 (MCP-1), IL-6, IL-1*β*, and TNF-*α* in the aorta of mice exposed to tobacco for a long period of time could be reduced to achieve the purpose of vascular protection [84].

## 7. Indole Alkaloid

Indole alkaloids, mainly derived from tryptophan, are the largest and most complex alkaloids. Indole alkaloids are divided into four groups: simple indoles, tryptophan indole, hemiterpenoid indoles, and monoterpene indoles.

### 7.1. Rhynchophylline

Rhynchophylline, a kind of monoterpene indole alkaloids, is the highest in the content of *Uncaria rhynchophylla* (Miq.) Miq. ex Havil. [85]. It has therapeutic effects on epilepsy, hypertension, and Parkinson's diseases. Rhynchophylline can significantly increase the pain threshold of mice and inhibit auricle swelling by reducing capillary permeability caused by xylene [86]. In vitro, 5 *μ*g/*μ*L of rhynchophylline can inhibit the production of IL-1*β* and TNF-*α* in dopaminergic neurons and glial cells induced by lipopolysaccharide, so as to protect neurons. Rhynchophylline can also inhibit the phosphorylation of p38 MAPK, then block the nuclear translocation of NF-*κ*B, and inhibit its transcriptional function, so as to inhibit the vascular endothelial cell damage caused by intermittent hypoxia [87].

### 7.2. Brucine

Brucine, a kind of monoterpene indole alkaloids extracted from the mature seeds of *Strychnos nux-vomica* Linn., has therapeutic effects on myasthenia gravis, rheumatoid arthritis, and hemiplegia caused by cerebrovascular diseases [88]. Brucine can inhibit auricle swelling caused by xylene in mice. Oral administration of 3 mg/kg of brucine can inhibit adjuvant arthritis in rats and reduce the concentrations of NO and NOS in serum, thus reducing the destruction of articular cartilage and protecting the function of chondrocytes [89]. In myasthenia gravis model rats, brucine can not only reduce the serum level of acetylcholine receptor antibody and IL-6, but also inhibit the activation of T cells and B cells.

## 8. Summary

As people learn more and more about nature, Chinese medical herbs have become important sources for the creation of new medicines. The purpose of researches on the anti-inflammatory effects and mechanisms of alkaloids in Chinese materia medica is to find new anti-inflammatory drugs with high selectivity and strong effect and improve the effectiveness, safety, and economy of the current clinical treatment in rheumatoid arthritis, ankylosing spondylitis, and other rheumatic immune diseases.

There are many side effects of drugs currently used in the treatment of rheumatic immune diseases. Nonsteroidal anti-inflammatory drugs can increase the risk of gastrointestinal bleeding. Glucocorticoids can not only raise blood sugar and blood pressure but also aggravate osteoporosis. Immunosuppressive agents can give rise to fungal or atypical pathogen infections. Alkaloids in Chinese materia medica have unique advantages in the treatment of rheumatic immune diseases. They exert anti-inflammatory effects through multiple targets, without adverse reactions mentioned above. Their anti-inflammatory mechanisms include stimulating the pituitary adrenal cortex axis, promoting the release of adrenal cortex hormones, inhibiting the release of inflammatory mediators, interleukins, and tumor necrosis factors, and regulating the level of nitric oxide, the expression of cytokine mRNA, and so on [90].

Anti-inflammatory alkaloids can be mainly divided into the following categories: isoquinoline alkaloids, indole alkaloids, pyridine alkaloids, terpenoid alkaloids, organic amine alkaloids, etc. This article summarizes anti-inflammatory mechanisms of alkaloids in Chinese materia medica, in order to provide a reference for screening active ingredients with anti-inflammatory effects and finding new therapeutic targets. However, the studies above are limited to animal experiments, and some of their mechanisms need further study. We will continue to explore the anti-inflammatory effects of alkaloids in Chinese materia medica in clinical practice and contribute our efforts to the development of new drugs with anti-inflammatory effects.
